# Responses of a native plant species from invaded and uninvaded areas to allelopathic effects of an invader

**DOI:** 10.1002/ece3.5195

**Published:** 2019-05-03

**Authors:** Anne Lyytinen, Leena Lindström

**Affiliations:** ^1^ Department of Biological and Environmental Science, Centre of Excellence in Biological Interactions Research University of Jyväskylä Jyväskylä Finland

**Keywords:** *Anthriscus sylvestris*, evolutionary response, invasion, local adaptation, *Lupinus polyphyllus*, native plant

## Abstract

Invaders exert new selection pressures on the resident species, for example, through competition for resources or by using novel weapons. It has been shown that novel weapons aid invasion but it is unclear whether native species co‐occurring with invaders have adapted to tolerate these novel weapons. Those resident species which are able to adapt to new selective agents can co‐occur with an invader while others face a risk of local extinction. We ran a factorial common garden experiment to study whether a native plant species, *Anthriscus sylvestris*, has been able to evolve a greater tolerance to the allelochemicals exerted by the invader, *Lupinus polyphyllus*. *Lupinus polyphyllus* produces allelochemicals which potentially act as a novel, strong selective agent on *A. sylvestris*. We grew *A. sylvestris* seedlings collected from uninvaded (naïve) and invaded (experienced) sites growing alone and in competition with *L. polyphyllus* in pots filled with soil with and without activated carbon. Because activated carbon absorbs allelochemicals, its addition should improve especially naïve *A. sylvestris* performance in the presence of the invader. To distinguish the allelochemicals absorption and fertilizing effects of activated carbon, we grew plants also in a mixture of soil and fertilizer. A common garden experiment indicated that the performances of naïve and experienced *A. sylvestris* seedlings did not differ when grown with *L. polyphyllus*. The addition of activated carbon, which reduces interference by allelochemicals, did not induce differences in their performances although it had a positive effect on the aboveground biomass of *A. sylvestris*. Together, these results suggest that naïve and experienced *A. sylvestris* plants tolerated equally the invader *L. polyphyllus* and thus the tolerance has not occurred over the course of invasion.

## 1 INTRODUCTION

The ever increasing number of native species is challenged by new competitors, invasive species, whose number has increased since 1800 (Seebens et al., 2017). When a species invades a new area, it may bring in addition to competition new selection pressures which can be detrimental to native species. The invasions may be ameliorated if natives are capable of adapting to the presence of an invader. Studies have demonstrated the ability of native species to evolve as a response to invasion (reviews by Strauss, Lau, and Carroll (2006) and Oduor (2013)), but the absence of evolutionary responses has also been reported (Goergen, Leger, & Espeland, 2011; Lau, 2006; Mealor & Hild, 2007). Native populations can respond evolutionarily if they, for example, possess enough genetic variation, selection pressure is strong and consistent, and gene flow between uninvaded and invaded native populations is limited (Leger & Espeland, 2010; Strauss et al., 2006). In addition to the evolutionary factors, ecological factors, such as multiple species interactions (Lau, 2006), may limit the evolutionary capacity (review by Lau and terHorst (2015)).

Allelopathy refers to the negative and positive effects of biochemicals, so‐called allelochemicals, produced by an organism on another organism (Rice, 1984). In the context of invasion, the role of allelochemicals has been studied mainly from the perspective of invaders as a potential explanation for their superiority in an invaded area. The novel weapon hypothesis states that resident plants are negatively affected by the biochemicals released by an invader (i.e., allelopathic effects) to which they are not adapted (Callaway & Aschehoug, 2000). The fate of resident plants is not necessarily a stable state if they can evolve to tolerate invaders’ allelochemicals (Callaway, Ridenour, Laboski, Weir, & Vivanco, 2005), making coexistence with an invader more likely.

One of the successful invasive plant species is a garden lupine, *Lupinus polyphyllus*, which has been introduced from North America into Europe in the 1800s (Fremstad, 2010). In Europe, it causes environmental impacts (Rumlerová, Vilà, Pergl, Nentwig, & Pyšek, 2016), such as reduced plant and insect species richness (Ramula & Pihlaja, 2012; Valtonen, Jantunen, & Saarinen, 2006). Allelochemicals may be one of the factors contributing to its competition superiority and invasion success. *Lupinus* species, like *L. polyphyllus* (see e.g., Wink & Hartmann, 1981), contains quinolizidine alkaloids which they exudes into the soil from their roots (Wink, 1983). The major alkaloid is lupanine (Wink, Witte, Hartmann, Theuring, & Volz, 1983), which inhibits seed germination and seedling growth of interspecific neighbors (Muzquiz, de la Cuadra, Cuadrado, Burbano, & Calvo, 1994). Alkaloids can also be released from dead plant tissue. In *L. polyphyllus*, the allelopathic effects of litter seem to be minor compared to that of the root exudates. Litter of *L. polyphyllus* can reduce germination and increase germination time of native species but on the other hand, it increases seedling biomass, potentially due to the nutrients released through decomposition (Loydi, Donath, Eckstein, & Otte, 2015).

Although *L. polyphyllus* often forms monospecific stands, there are some species which are able to coexist with it such as the cow parsley, *Anthriscus sylvestris* (Valtonen et al., 2006). Our previous research showed that at least some *A. sylvestris* populations have evolved an increased competitive ability against *L. polyphyllus* (A. Lyytinen & L. Lindström, unpublished data). As native species are naïve to the allelochemicals produced by *L. polyphyllus*, the tolerance of remnant species to these allelochemicals may have increased in response to selection caused by *L. polyphyllus*. We set up an experiment to test whether *A. sylvestris* has adapted to allelochemicals released by *L. polyphyllus* into the soil. We collected *A. sylvestris* seedlings from uninvaded and invaded sites and grew them either alone or in competition with *L. polyphyllus*. We also manipulated the soil conditions by adding activated carbon, which absorbs allelochemicals. If local adaptation to invader chemicals has taken place, *L. polyphyllus* is expected to decrease the performance of *A. sylvestris* with no experience with *L. polyphyllus* compared to those conspecific individuals that have experienced invasion as they are naïve to the allelopathic chemicals of the invader. In the presence of activated carbon, this difference in performances is expected to be reduced because activated carbon should increase especially the performance of the most susceptible individuals. To assess the allelochemicals absorption and fertilizing effects of activated carbon, plants were also grown in a soil mixed with fertilizer.

## 2 MATERIALS AND METHODS

**Figure 1:**
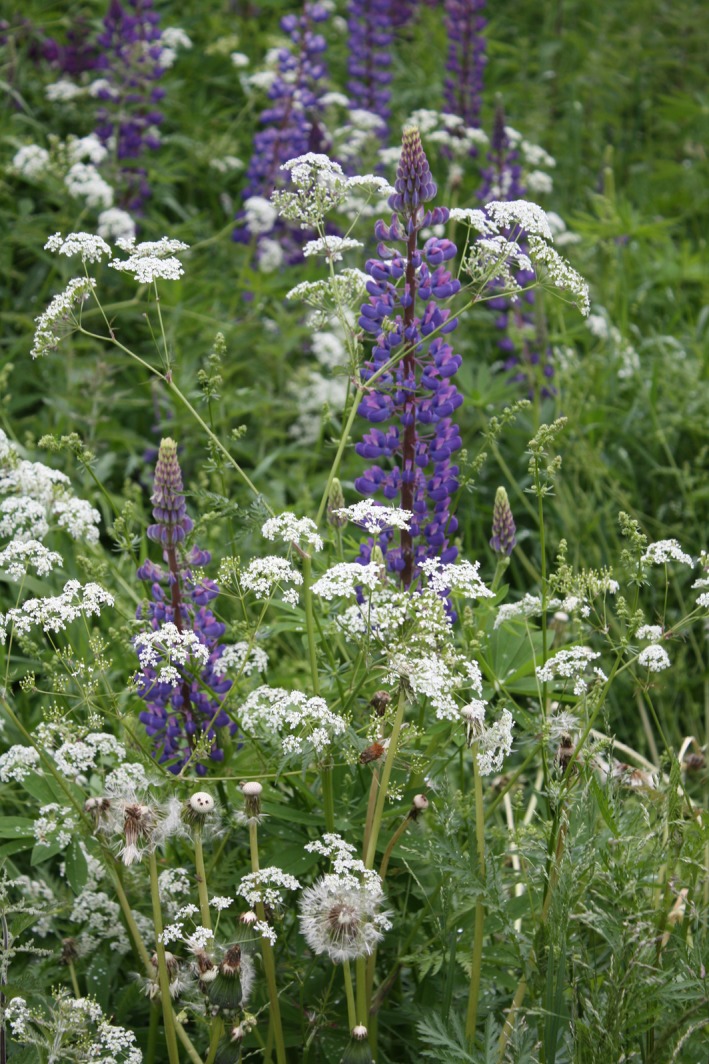
A mixed population of *Anthriscus sylvestris* and the invader *Lupinus polyphyllus*.

Seedlings of cow parsleys, *A. sylvestris*, were collected from three sites invaded (hereafter experienced) by a garden lupine, *L. polyphyllus* (Figure 1), and from three uninvaded sites (hereafter naïve) in Central Finland (Jyväskylä, 62°N, 25°E). *Anthriscus sylvestris* was planted together with *L. polyphyllus* in 3 L pots (diameter: 16 cm, tall: 12 cm) filled with a 2.5 L of 1:1 mixture of potting soil (Kekkilä Oy, viljelyseos) and sand. Both *A. sylvestris* and *L. polyphyllus* were also planted alone. Plants grown alone and in pairs were assigned into the three soil treatments: control, activated carbon, and fertilizer treatment (Table 1). Activated carbon (20 ml/L of soil, Merck KGaA) was mixed with the substrate to ameliorate the allelopathic effects of *L. polyphyllus*. As a control for the activated carbon treatment, we added fertilizer (0.4 g per pot, Kekkilä, puutarhalannoite, NPK 9‐4‐13) to another set of pots. This allowed us to distinguish the absorption of allelochemicals and fertilizing effects of activated carbon (Weißhuhn & Prati, 2009). Pots were placed randomly in a common garden at the University of Jyväskylä. The height of the stem was measured every second week until the growth was levelled off. After each measurement, the order of pots was again randomized. After 14 weeks, the stem was cut at the height of 3 cm from ground level, dried at 48°C for 7 days and weighed.

**Table 1:** The number of plants in each treatment at the beginning of the first and second growing season. In the second year, only those plant pairs were included where both were alive after the first winter. Plants were collected from uninvaded (naïve) and invaded (experienced) sites*. Anthriscus sylvestris *plants, which were grown alone or in competition with the invader *L. polyphyllus*, were allocated to soil treatments: control, activated carbon, and fertilizer

			Soil treatment 2013/2014		
Species	Invasion history	Competition treatment	Control	AC	Fertilizer
*A. sylvestris*	Naïve	Alone	17/11	17/13	17/16
	Experienced	Alone	16/9	16/13	15/15
	Naïve	Competition	13/6	13/5	13/3
	Experienced	Competition	15/6	16/2	15/3

For overwintering, pots were dug into the soil so that the pot rim was at the soil level. In the following spring, pots were dug up and placed in a common garden. Only those pairs where both plants were alive were included in the experiment (Table 1). Similarly, as in the first growing season, the height of the stem was measured every second week until the growth was levelled off when the stems and roots were harvested. After drying at 48°C, the stems and roots were weighed. In total, the experiment lasted 448 days (two growing seasons).

### 2.1 Data analysis

We performed separate tests for competition treatments (grown alone, grown together with *L. polyphyllus*). To test whether plant height growth pattern of *A. sylvestris* differs with the invasion history or soil treatment in the first growing season, we performed a repeated 2‐way ANCOVA with the height measurements as different factor levels and the invasion history (naïve, experienced) and soil treatment (control, activated carbon, fertilizer) as fixed factors and a root length of a seedling as a covariate. For the data from the second growing season, the model included also the initial shoot height of the seeding as a covariate. To test which of the factors affect the final shoot and root dry weight, and root‐shoot ratio, we performed separate Generalized Linear Models where the invasion history and soil treatment were fixed factors, and a root and shoot length of a seedling were covariates. The pairwise comparisons were tested with least significant difference (LSD). Survival from the beginning of the experiment to the following spring was analyzed with Binary logistic regression. All analysis were performed with SPSS Statistics 24.

## 3 RESULTS

### 3.1 Height growth pattern of A. sylvestris

**Figure 2:**
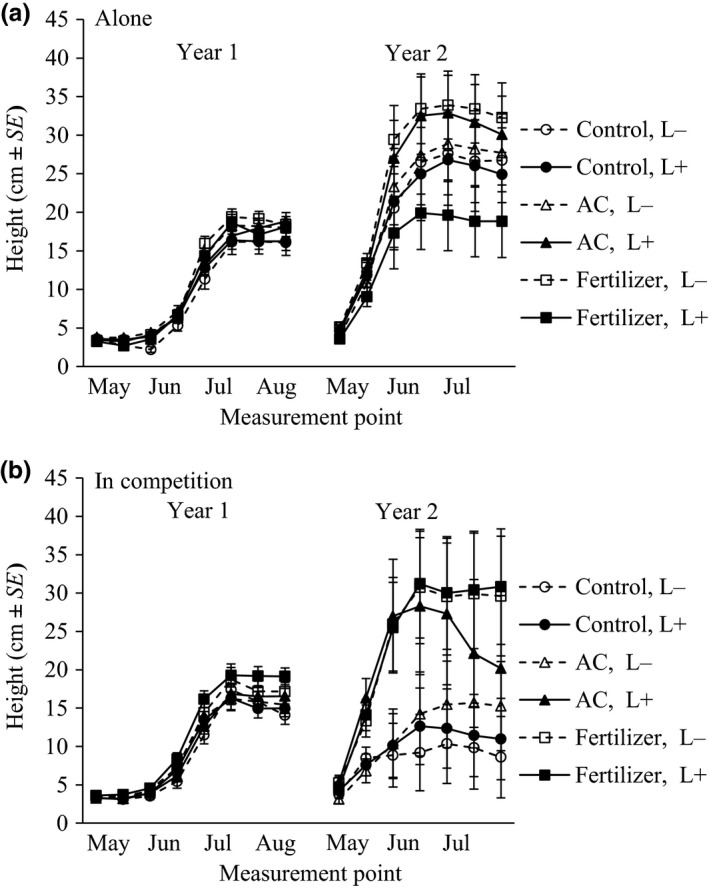
The height growth pattern of *A. sylvestris* (estimated marginal means ± *SE*) from uninvaded (dashed line, L−) and invaded (solid line, L+) sites in the first and second growing season. *Anthriscus sylvestris* plants were grown (a) alone or (b) in competition with the invader *L. polyphyllus* in pots filled with a mixture of substrate and activated carbon (AC, triangle) or fertilizer (square). Control (circle) plants were grown without activated carbon and fertilizer

When *A. sylvestris* plants were grown alone, there was a trend that the soil treatment resulted in differences in the growth pattern only in the first growing season and independent of the invasion history (Figure 2a, Table 2). Those plants which were grown in substrate with fertilizer showed a tendency of higher growth pattern than control plants (Least Significant Difference (LSD): *p* = 0.046). The addition of activated carbon did not induce differences in growth pattern compared to control plants (LSD: *p* = 0.074) or to the addition of fertilizer (LSD: *p* = 0.860). In both growing seasons, invasion history and seedling root length (covariate) did not affect the growth pattern.

**Table 2:** Results of the repeated 2‐way ANCOVA testing for effects of the invasion history (naïve, experienced) and soil treatment (control, activated carbon, fertilizer) on the growth pattern in *A. sylvestris* plants which were grown alone or in competition with the invader *L. polyphyllus*. A shoot and root length of a seedling were covariates

	Year 1			Year 2		
Source	*df*	*F*	*p*	*df*	*F*	*p*
Alone						
Covariate, shoot, seedling				1	3.063	0.085
Covariate, root, seedling	1	0.924	0.339	1	0.001	0.970
Invasion history	1	0.249	0.619	1	0.784	0.379
Soil	2	2.448	**0.093**	2	0.299	0.743
Invasion history × Soil	2	0.753	0.474	2	1.975	0.146
Error	80			69		
In competition						
Covariate, shoot, seedling				1	0.831	0.375
Covariate, root, seedling	1	0.111	0.740	1	1.922	0.184
Invasion history	1	0.504	0.480	1	0.890	0.359
Soil	2	3.208	**0.046**	2	5.273	**0.017**
Invasion history × Soil	2	0.305	0.738	2	0.452	0.644
Error	74			17		

All significant *p* < 0.05 values are given as bold.

When *A. sylvestris* plants were grown in competition with *L. polyphyllus*, soil treatment resulted in differences in the growth patterns in both the first and the second growing season (Figure 2b, Table 2). The addition of fertilizer resulted in a higher grown pattern compared to the control treatment (LSD: *p* = 0.017 and *p* = 0.006, respectively). The addition of activated carbon did not induce differences compared to fertilizer (*p* = 0.072 and *p* = 0.199, respectively) or control treatments (*p* = 0.505 and *p* = 0.142, respectively). The effect of the soil treatment did not differ with the invasion history. The root or shoot length of a seedling did not affect the growth pattern.

### 3.2 The final biomass and root‐shoot ratio in A. sylvestris

**Figure 3:**
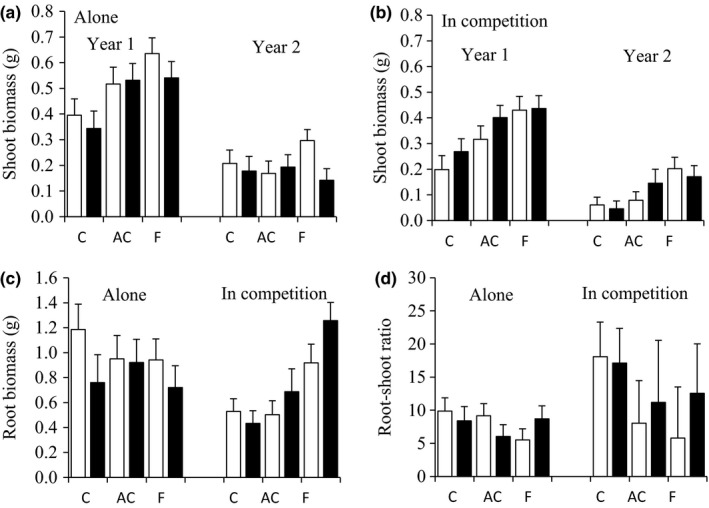
(a, b) The shoot biomass (estimated marginal means (g) + *SE*) in the first and second growing season, (c) root biomass, and (d) root‐shoot ratio of *A. sylvestris* plants. *A. sylvestris* plants from uninvaded (white bars) and invaded sites (black bars) were grown alone or in competition with the invader *L. polyphyllus* in substrate with activated carbon (AC) or fertilizer (F) for 2 growing seasons. Control (C) plants were grown in substrate without activated carbon and fertilizer

In the first growing season, the invasion history by soil treatment interaction and the main effect of invasion history (naïve, experienced) on shoot biomass in alone grown *A. sylvestris* were not significant (Table 3, Figure 3a). In contrast, shoot biomass differed with the soil treatment. Activated carbon increased the shoot biomass by 42% compared to the controls (LSD: *p* = 0.018). Fertilizer increased biomass even more: 59 % (*p* = 0.001). Fertilizer addition resulted in a similar biomass than activated carbon (alone: *p* = 0.320). The size of the seedling or the root length of the seedling did not affect the biomass. At the end of the second growing season, there were no main or interactive effects of the invasion history and soil treatment on the shoot biomass (Figure 3a), root biomass (Figure 3c), or root‐shoot ratio (Figure 3d) (Table 3). Only the shoot length of the seedling affected shoot biomass and the root‐shoot ratio. The longer the shoot was, the heavier the shoot biomass and the smaller the ratio were.

**Table 3:** Results of General Linear Model testing for effects of the invasion history (naïve, experienced) and soil treatment (control, activated carbon, fertilizer) on the final biomass of the shoot and root, and root‐shoot ratio in *A. sylvestris* grown alone or in competition with the invader *L. polyphyllus*. A shoot and root length of a seedling were covariates. A significant factor is marked in bold font

		Alone						In competition					
		Year 1			Year 2			Year 1			Year 2		
Trait	Source	*df*	*χ* ^2^	*p*	*df*	*χ* ^2^	*p*	*df*	*χ* ^2^	*p*	*df*	*χ* ^2^	*p*
Shoot biomass	Covariate, shoot, seedling	1	0.962	** 0.327**	1	4.089	**0.043**	1	10.932	**0.001**	1	4.055	**0.044**
	Covariate, root, seedling	1	0.726	0.394	1	0.132	0.716	1	1.018	0.313	1	1.967	0.161
	Invasion history	1	0.688	0.407	1	1.742	0.187	1	1.575	0.210	1	0.044	0.834
	Soil	2	12.128	**0.002**	2	0.717	0.699	2	15.022	**0.001**	2	12.792	**0.002**
	History × Soil	2	0.741	0.690	2	4.042	0.133	2	0.659	0.719	2	1.465	0.481
Root biomass	Covariate, Shoot, seedling				1	0.229	0.633				1	0.103	0.748
	Covariate, Root, seedling				1	3.737	0.053				1	2.546	0.111
	Invasion history				1	2.073	0.150				1	1.682	0.195
	Soil				2	0.618	0.734				2	22.834	**<0.001**
	History × Soil				2	0.967	0.617				2	3.319	0.190
Root‐shoot ratio	Covariate, Shoot, seedling				1	5.566	**0.018**				1	0.023	0.878
	Covariate, Root, seedling				1	0.002	0.966				1	0.026	0.872
	Invasion history				1	0.099	0.754				1	0.270	0.604
	Soil				2	1.136	0.567				2	2.409	0.300
	History × Soil				2	3.230	0.199				2	0.374	0.829

When *A. sylvestris* plants were grown in competition with *L. polyphyllus* for one growing season, the invasion history by soil treatment interaction and the main effect of invasion history on shoot biomass were not significant (Table 3, Figure 3b). On average, activated carbon increased the shoot biomass by 53% compared to the controls (*p* = 0.013), producing plants with equal biomass as the addition of fertilizer (*p* = 0.143). Fertilizer, however, increased biomass even more than the addition of activated carbon, by 86% compared to the controls (*p* < 0.001). Unlike the root length of the seedling, the size of the seedling affected shoot biomass. The longer the shoot length of the seedling was, the heavier the shoots were. The results from the second growing season were consistent with the first growing season. Neither the invasion history by soil treatment interaction nor the invasion history significantly affected the shoot and root biomass whereas soil treatment did. *A. sylvestris* plants, which grew in fertilized substrate, had a higher shoot (LSD: *p* < 0.001, increase 112%, Figure 3b) and root biomass (*p* < 0.001, increase 126%, Figure 3c) than control plants. The addition of fertilizer also produced *A. sylvestris* plants with greater root biomass than the addition of activated carbon (*p* = 0.001), but such difference was not observed in shoot biomass (*p* = 0.106). The shoot (*p* = 0.120) and root biomass (*p* = 0.372) of plants grown with and without activated carbon were equal. Root‐shoot ratio was not affected by invasion history, soil treatment, their interaction, or covariates (the shoot and root length of the seedling, Figure 3d).

### 3.3 Survival

When *A. sylvestris* plants were grown alone, invasion history did not affect survival from the beginning of the experiment to the following spring (Binary logistic regression: Wald = 0.008, *df* = 1, *p* = 0.930; naïve: 78%; experienced: 79%) whereas the soil treatment caused differences in survival (Wald = 8.821, *df* = 2, *p* = 0.012). The addition of fertilizer improved survival compared to the control plants (97% vs. 61%) (Wald = 7.784, *df* = 1, *p* = 0.005) unlike the addition of activated carbon (79% vs. 61%) (Wald = 2.521, *df* = 1, *p* = 0.112). Furthermore, survival tended to be higher for plants grown in soil with fertilizer than for those grown with activated carbon (Wald = 3.712, *df* = 1, *p* = 0.054).

When *A. sylvestris* plants were grown in competition with *L. polyphyllus*, survival did not differ either with invasion history (Wald = 0.028, *df* = 1, *p* = 0.867; naïve: 80%; experienced: 78%) or soil treatment (Wald = 2.984, *df* = 2, *p* = 0.225; control: 75%, activated carbon: 90%, fertilizer: 71%).

## 4 DISCUSSION

Some plant invaders possess allelochemicals which reduce the performance of resident species in the invaded communities. However, resident species can evolve an increased tolerance to allelopathic compounds and instead of going locally extinct they can persist with an invader. To test the tolerance of native species against an invader, we compared performance of a native plant species, *A. sylvestris* from uninvaded and invaded sites and grown the plants together with an invader. Contrary to the expectation, the naïve and experienced *A. sylvestris* plants had equal shoot and root biomass as well as height growth patterns and shoot‐root ratio when grown in competition with the invader *L. polyphyllus*. Furthermore, the addition of activated carbon to the substrate, which absorbs allelochemicals and thereby neutralizes their effects, did not increase the performance of naïve *A. sylvestris* plants compared to naïve individuals grown in the substrate without activated carbon. These results point to the conclusion that exposure to invasion by *L. polyphyllus* has not resulted in an increased tolerance to *L. polyphyllus* allelochemicals in *A. sylvestris*. We, however, did detect some indication of allelopathic effects of *L. polyphyllus* on *A. sylvestris*.

There are several potential explanations for the absence of differences in performances between naïve and experienced *A. sylvestris*. First, the strength of the selection might not been strong enough to result in local adaptation (Kawecki & Ebert, 2004). In our experiment, activated carbon had a positive effect on above ground biomass of the native plants in the first growing season, indicating that *L. polyphyllus* may have had some allelopathic effects on *A. sylvestris*. On the other hand, the similar survival of naïve and experienced plants both with and without activated carbon addition suggests that either *L. polyphyllus* do not exude allelochemicals in high quantities through roots or they do not create a selection pressures on growing *A. sylvestris*. Second, even though the selection would have been strong enough, the gene flow from uninvaded areas might have hindered adaptation if its homogenizing effect has been stronger than the strength of selection (Kawecki & Ebert, 2004). As *A. sylvestris* is a common and abundant plant in Finland in overlapping areas with *L. polyphyllus* (Lampinen & Lahti, 2016) and its pollinators travel long distances (Rader, Edwards, Westcott, Cunningham, & Howlett, 2011), gene flow between naïve and experienced populations is likely. Third, it is also possible that invasion has been recent and there has not been enough time to allow evolutionary changes. Because of imperfect knowledge of invasion history of *L. polyphyllus*, we are able only to estimate the length of the period of association. The first reports of wild stands of *L. polyphyllus* in Finland are from 1895 (Fremstad, 2010) and they reached the present collection sites, Central Finland, by 1970s (Lampinen & Lahti, 2016). Based on this, we can estimate the maximum age of *L. polyphyllus* populations to be 40 years. Other plants have been reported to adapt to novel allelochemicals even in a shorter time period (20–30 years) (Callaway et al., 2005) although the length of the association also has based on estimated invasion times.

The lack of differences among naïve and experienced *A. sylvestris* plants as a response to *L. polyphyllus* could also indicate that roots may not exude allelochemicals in large quantities to have measurable allelopathic effects (but see Wink, 1983). The majority of the studies examining alkaloids in *Lupinus* species has focused on the seeds. As alkaloids are synthetized mainly in the green parts of *L. polyphyllus* (Wink, Hartmann, & Witte, 1980), it is possible that adding withering leaves could have had larger effect on the growth of *A. sylvestris*.

Adding activated carbon to the substrate resulted in the improved performance of *A. sylvestris* plants when grown in competition with *L. polyphyllus* but only in the first growing season. Furthermore, activated carbon increased the above ground biomass relatively more in plants grown in competition with *L. polyphyllus* (a 53% increase compared to controls) than that of those grown alone (42%). This indicates that allelochemicals released by *L. polyphyllus* contributed to the decreased biomass of *A. sylvestris* plants. The addition of fertilizer had even a more positive effect on the aboveground biomass of *A. sylvestris* than activated carbon (over an 86% increase). Furthermore, the addition of fertilizer doubled the belowground biomass for *A. sylvestris*. The results suggest that competition for resources was a more important mechanism underlying the harmful impacts of the presence of *L. polyphyllus* on the biomass of *A. sylvestris* than allelopathy. Based on our experiment, we cannot conclude for which resources plants competed. Due to symbiotic nitrogen fixation in *L. polyphyllus*, it is unlike that there was shortage of nitrogen. Also, in other native‐invader interactions, suppression of performance of native species has accounted more for resource competition than allelopathy (Nickerson & Flory, 2015) but also opposite results have been reported (Ridenour & Callaway, 2001).

In the second growing season, the addition of activated carbon to the substrate did not alter the effects of the presence of *L. polyphyllus* on *A. sylvestris*, suggesting the absence of allelopathic effects. One possible explanation for the dissimilar results compared to the first growing season is the reduced sample size. The mortality among the pairs which were exposed to competition from *L. polyphyllus* was high, diminishing the power of statistical tests to detect differences.

As *A. sylvestris* appeared to be relatively tolerant to *L. polyphyllus* allolechemicals, one might ask whether it has experienced competition from a species which also contains the same alkaloids as *L. polyphyllus*. The main quinolizidine alkaloid in *L. polyphyllus* is lupanine (Wink et al., 1983) which is not known to occur in any other species growing wild in Finland than *L. polyphyllus* (Aniszewski, 2007). Thus, naïve *A. sylvestris* has most likely not been exposed earlier to it.

Although we found some indications of allelopathic effects, we did not find evidences that experienced *A. sylvestris* plants have evolved an increased tolerance to the *L. polyphyllus* allelochemicals. However, there was among individual variation in aboveground biomass, indicating potential for evolution. On the basis of a greater positive effect of addition of fertilizer compared to activated carbon, we can infer that competition for resources is more important factor than allelopathy behind the harmful effects of *L. polyphyllus* on *A. sylvestris*.

## CONFLICT OF INTEREST

Authors declare no competing interest.

## AUTHOR CONTRIBUTIONS

AL and LL conceived the ideas and designed methodology; AL collected and analyzed the data; AL and LL led the writing of the manuscript. Both authors contributed critically to the drafts and gave final approval for publication.

## DATA ACCESSIBILITY

When the paper will be published, our data will be included in the University of Jyväskylä open access database JYX Digital Repository (https://jyx.jyu.fi/handle/123456789/63516) according to the guidelines of Ecology and Evolution and the University of Jyväskylä.
